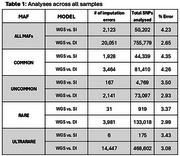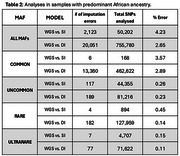# Traditional vs. Double‐round Genotype Imputation in multi‐ethnic Cohorts

**DOI:** 10.1002/alz70855_107394

**Published:** 2025-12-24

**Authors:** Sarwan Ali, Basilio Cieza, Giuseppe Tosto

**Affiliations:** ^1^ Columbia University, New York, NY, USA; ^2^ Department of Neurology, College of Physicians and Surgeons, Columbia University, and the New York Presbyterian Hospital, New York, NY, USA

## Abstract

**Background:**

Imputation is still a crucial technique in genomic studies to infer untyped variants, enhancing the coverage and power of genome‐wide association studies. However, its accuracy can vary, especially for rare variants and across populations. Using genotype data from ADSP (Alzheimer's Disease sequencing Project), we conducted I) traditional imputation with a single round of genotype imputation (“SI”) and II) two (or more) rounds of imputation (“DI”) on data progressively passed through quality control and again imputed. We tested the performance of either approach by estimating the amount of imputation errors using whole genome sequencing data (WGS) from ADSP as gold standard.

**Method:**

For 196 Caribbean Hispanics, we estimated the error rates only in SNPs within chromosome 1 imputed at optimal quality (R^2≥ 80%) and across minor allele frequency (MAF) brackets: common (MAF ≥ 0.05), uncommon (0.01 ≤ MAF < 0.05), rare (0.001 ≤ MAF < 0.01), ultra‐rare (MAF < 0.001) and overall. We tested the entire sample and then separately for individuals with ≥50% African genetic ancestry (AFR).

**Result:**

Overall, DI showed significant lower error rates compared to SI (2.65% vs. 4.23%, Wilcoxon *p*‐value < 0.001). This result was consistent across MAF (Table 1), in rare (2.99% vs. 3.37%), uncommon (2.93% vs. 3.50%), and ultra‐rare variants (3.08% vs. 3.43%). In individuals with predominant AFR, imputation errors were more frequently observed compared to those with low AFR, especially for rare and ultra‐rare variants; nevertheless, DI maintained significant lower error rates across all MAF categories (Table 2).

**Conclusion:**

Our findings demonstrate that multiple rounds of imputation generally outperform the traditional single one in terms of accuracy, particularly for rare and ultra‐rare variants. This improvement is confirmed in groups that have shown higher error rates after traditional imputation, such as individuals with predominant African ancestry (*Sariya et al. 2019*). These results highlight a valuable and easy approach for enhancing the quality of imputed data across populations, which could lead to more robust genetic association studies.